# Fluid biopsies for *in vitro* fertilization: Non-invasive innovations (Review)

**DOI:** 10.3892/mi.2025.290

**Published:** 2025-12-16

**Authors:** Senem Aslan Öztürk, Nergis Özlem Kiliç, Duygu Kütük, Çağri Öner

**Affiliations:** 1 Department of Histology and Embryology, Faculty of Medicine, Maltepe University, 34857 İstanbul, Turkey; 2 Department of Medical Laboratory Techniques, Vocational School, İstanbul Atlas University, 34408 İstanbul, Turkey; 3 Department of *In-vitro* Fertilization, Medical Park Hospital, 35230 İzmir, Turkey; 4 Department of Medical Biology, Faculty of Medicine, Kırklareli University, 39100 Kırklareli, Turkey

**Keywords:** embryo culture medium, extracellular vesicle, follicular fluid, non-invasive biomarkers, reproductive diagnostics

## Abstract

Non-invasive fluid biopsies are emerging as a promising, low-risk complementary method to traditional diagnostic approaches in reproductive biology, allowing for repeated sampling throughout different stages of assisted reproductive technology. These approaches hold significant potential, not only for clinical diagnosis, but also for personalized medicine. The present review summarizes recent findings on key biomolecular components found in accessible body fluids, such as follicular fluid, embryo culture medium, uterine secretions and saliva, namely cell-free nucleic acids, extracellular vesicles and microRNAs. These biomarkers indicate critical cellular events, such as apoptosis, oxidative stress, mitochondrial dysfunction and intercellular signaling pathways. Special emphasis is placed on the molecular profile of granulosa and cumulus cells, which are essential for oocyte maturation and have strong predictive value for fertilization potential and subsequent pregnancy outcomes. Their molecular signatures provide critical information about the developmental competence of the oocyte and early embryo. However, limitations such as maternal contamination, mosaicism and variable compliance, along with a lack of guideline-level approval, currently restrict their routine clinical use. These non-invasive samples are valuable complements to current invasive methods, providing information about chromosomal competence and embryo viability without compromising embryo integrity. Overall, the current evidence highlights the potential of fluid-based biomarker discovery to improve the outcomes of *in vitro* fertilization (IVF) and support the development of personalized, sustainable fertility treatments. The present review aimed to summarize and critically evaluate developments in non-invasive liquid biopsy techniques in IVF, highlight their clinical applications for improving IVF outcomes, and examine the molecular profile of granulosa and cumulus cells as determinants of oocyte and embryo quality. The fundamental hypothesis is that non-invasive liquid biopsies can serve as effective, low-risk alternatives to traditional invasive diagnostic approaches, minimizing harm to patients and embryos, while improving embryo selection, preimplantation genetic testing and overall IVF success.

## 1. Introduction

Non-invasive fluid biopsies in *in vitro* fertilization (IVF) aim to address several critical clinical gaps that limit the efficacy and safety of current reproductive diagnostic methods (1,2). Traditional invasive techniques, such as embryo biopsy for genetic testing, carry risks of damaging embryos and causing patient discomfort, and are typically limited to single-time sampling, which restricts the ability to monitor dynamic changes throughout assisted reproductive technology (ART) cycles (3,4). These methods also rely heavily on subjective morphological assessments for embryo selection, which may not accurately predict embryo viability or genetic competence (5,6). Additionally, invasive genetic testing can be affected by maternal DNA contamination and require specialized laboratory resources, rendering it less accessible and potentially less reliable (7,8). There is also a lack of standardized protocols, leading to variability in results (9,10), and valuable biological materials such as granulosa and cumulus cells are often discarded, despite containing crucial molecular information (11). By enabling the repeated, low-risk sampling of accessible body fluids and providing objective molecular biomarkers, non-invasive fluid biopsies provide a promising solution to improve embryo selection, enhance genetic testing accuracy, and support more personalized and sustainable reproductive therapies in IVF (10).

Current invasive methods in IVF, such as embryo biopsy for preimplantation genetic testing (PGT), present a number of limitations and risks (12). These procedures can potentially harm the embryo, reduce its viability and lead to lower implantation and pregnancy rates (13). Additionally, invasive sampling is uncomfortable for patients and is typically restricted to a single time point, which limits the ability to monitor dynamic changes throughout the ART cycle. The reliance on subjective morphological assessments for embryo selection further compounds the issue, as these visual evaluations may not accurately reflect the embryo's genetic competence or developmental potential (14). Invasive genetic testing also faces challenges, such as maternal DNA contamination and the need for specialized laboratory resources, rendering it less accessible and at times, less reliable (7). Non-invasive fluid biopsy alternatives, by contrast, enable the repeated, low-risk sampling of accessible body fluids, such as follicular fluid, embryo culture media and uterine secretions (9,15). These approaches provide objective molecular biomarkers, such as cell-free nucleic acids, extracellular vesicles and microRNAs (miRNAs/miRs), that can more accurately predict embryo quality and viability (10,16). By minimizing harm to embryos and patients, and providing more reliable, accessible and dynamic diagnostic information, non-invasive fluid biopsies have the potential to overcome the major limitations of current invasive methods and revolutionize fertility diagnostics and therapeutic decision-making in IVF (17,18).

The present review summarizes recent findings on key biomolecular components found in accessible body fluids, such as follicular fluid, embryo culture medium, uterine secretions and saliva, namely cell-free nucleic acids, extracellular vesicles and miRNAs.

## 2. About non-invasive fluid biopsy samples and related molecules

Non-invasive fluid biopsies play a crucial role in medical diagnosis and monitoring, providing advantages, such as the ease of collection, reduced patient discomfort and the potential for repeated sampling. Various body fluids, including urine, saliva, interstitial fluid and nasal secretions, have been explored for their biomarker content to aid in the non-invasive detection of various diseases (19-21). These biomarkers include circulating tumor cells and trophoblastic cells, as well as more numerous, cell-free nucleic acids (cfNAs) such cell-free DNA (cfDNA), cell-free RNA (cfRNA) and circulating miRNAs. Furthermore, cfNAs not only circulate in isolation, but can also associate with protective protein complexes or be encapsulated in extracellular vesicles (EVs) (22).

cfNAs, including cfDNA and cfRNA originate from cultured cells, non-malignant somatic tissues, tumors, embryos, or fetuses and are released when cells undergo necrosis or apoptosis (23). cfNAs can be characterized by their length, physical size, surface molecules, electrical charge and density. cfDNA can also be detected in the blastocellular fluid of human embryos and the IVF culture medium used, allowing minimal and non-invasive genetic testing, respectively (24). In fact, the presence of mitochondrial DNA (mtDNA) in the culture medium of the embryo has been associated with embryo lysis caused by apoptosis or necrosis. Stigliani *et al* (25) found a strong association between mtDNA levels in the culture medium and human embryo fragmentation, which suggests that higher mtDNA concentrations indicate cellular distress and apoptosis in embryos. Furthermore, another study highlighted that the amount of DNA present in the culture medium is negatively associated with the competence of human embryos and clinical pregnancy outcomes, implying that the release of mtDNA may reflect the underlying embryonic competence (26).

## 3. Extracellular vesicles and their usage as non-invasive fluid samples

EVs are crucial mediators of intercellular communication, facilitating the exchange of biological signals among both prokaryotic and eukaryotic cells. These vesicles, which include exosomes, microvesicles and apoptotic bodies, vary in composition and size, ranging from 30 to 1,000 nm, and contain a variety of proteins, lipids and nucleic acids (27). These vesicles function as carriers of biomarkers and play essential roles in tissue regeneration, rendering them potential targets for therapeutic interventions in various diseases (28).

The implantation of the embryo is a critical step for a successful pregnancy and requires a complex interaction between the embryo and the endometrium. In a previous study investigating the involvement of EVs in embryo implantation and endometrial diseases, EVs were isolated from uterine fluid, cultured endometrial, epithelial/stromal and trophectodermal cells (2s). Endometrial, epithelial and stromal/decidual cell-derived EVs are covered by trophoblast cells, which regulate several gene sequences involved in adhesion, invasion and migration. Conversely, embryo-derived EVs are internalized by epithelial and immune cells of the endometrium for the biosensing and immunomodulation required for successful implantation. EVs have also been shown to play a role in infertility, recurrent implantation failure, endometriosis, endometritis and endometrial cancer (29). Further research will pave the way for the use of EVs as non-invasive ‘liquid biopsy’ tools for assessing endometrial health.

Ng *et al* (30) analyzed a panel of 227 endometrial exosomal miRNAs and demonstrated that a number of their target genes regulate key pathways involved in implantation. Exosomal miRNAs not only regulate key pathways, such as the VEGF pathway, Toll-like receptor pathway and Jak-STAT pathway, but also adhere to extracellular matrix-receptor interactions and junctions. Vilella *et al* (31) demonstrated that hsa-miR-30d derived from the endometrial exosome, when taken up by trophoblasts, increased the gene expression of integrin subunit alpha 7, integrin subunit beta-3 and cadherin 5 required for blastocyst implantation. Greening *et al* (32) further demonstrated that endometrial EVs increased their adhesiveness through the focal adhesion kinase signaling pathway when internalized by the trophectoderm.

## 4. Non-invasive fluid samples and preimplantation genetic testing

The use of PGT has expanded significantly in recent years, driven by advances made in genetic testing technologies and an increased understanding of genetic disorders. Major reproductive societies endorse PGT for various indications, including aneuploidy screening, HLA matching and the prevention of genetic diseases (33). The most common indication for PGT remains aneuploidy screening, particularly for couples with an advanced maternal age or those experiencing recurrent implantation failure (34). Despite its benefits, traditional PGT methods continue to face challenges, including the potential for damage to embryos and the need for specialized laboratory resources (1). While non-invasive PGT provides the potential to reduce embryo harm and patient risk, its current limitations, particularly maternal contamination, a low cfDNA yield, diagnostic variability, the lack of standardization and biological uncertainties, need to be critically addressed through further research and protocol optimization before it can reliably replace invasive methods in clinical IVF practice (2-4,35).

Kuznyetsov *et al* (7) demonstrated that combining spent embryo culture media and blastocoel fluid enhanced the quantity and quality of cfDNA available for aneuploidy testing, potentially improving the accuracy of genetic assessments. This approach minimizes the risks associated with invasive biopsies, while still providing valuable genetic information.

Moreover, Brouillet *et al* (8) conducted a systematic review, indicating that cfDNA in spent embryo culture medium could serve as a viable alternative to embryo biopsy for PGT, highlighting its potential to streamline the testing process and reduce costs. The analysis of EVs and miRNAs in non-invasive samples has also shown that they hold promise as additional biomarkers for embryo health and viability (36).

The integration of non-invasive testing could lead to higher implantation rates, reduced miscarriage rates and improved overall outcomes for couples undergoing IVF. Furthermore, advancements in next-generation sequencing and bioinformatics are expected to enhance the accuracy and reliability of non-invasive genetic testing methods, rendering them more accessible to a broader range of patients (33).

### Follicular fluid

Embryo selection procedures in IVF aim to identify high-quality embryos with the highest implantation potential. cfDNA of apoptotic granulosa cells is present in follicular fluid, which influences follicle maturation and oocyte growth *in vivo*. This biomarker can be used to sample the developmental competence of the contained oocyte during the oocyte retrieval phase of IVF treatment. Low levels of cfDNA in follicular fluid are significantly associated with a low embryo fragmentation rate and are indicative of high-quality embryos (37). Furthermore, extracellular mtDNA is actively released by granulosa cells into the follicular fluid in response to mitotic failure, and a low mtDNA content is associated with high oocyte developmental ability, and this allows for the determination of the viability of the embryo (38).

### Cumulus-oocyte complex (COC) and granulosa cells

The COC is involved in several key processes, including oocyte growth, metabolic regulation and cellular adhesion to the oocyte membrane. It also facilitates intercellular communication between the cumulus cells and the oocyte, which is essential for oocyte maturation and successful fertilization. Additionally, the COC is necessary for the expansion of the cumulus oophorous and plays a role in the spatial distribution of granulosa cells within the follicle (39). The complex also regulates gap junctions and cytoskeletal changes and influences polar body displacement after treatments to remove cumulus-corona cells in preparation for assisted reproductive methods (40). The COC is a dynamic structure that undergoes changes during *in vitro* culture, influenced by various factors, such as biological (e.g., age-related) and external (e.g., co-enzyme Q10 supplementation) conditions (41).

In cases of mitochondrial malfunction, it has been shown that cumulus cells surrounding the oocyte during development can increase the levels of cf-mtDNA in IVF culture media (42). Researchers are investigating the effects of mitochondrial dysfunction to predict the developmental competence and implantation potential of embryos, as well as to better understand embryo quality (43-45). Since the expression of specific genes in cumulus cells is linked to embryo potential and pregnancy outcomes, cumulus cell gene expression is a reliable indicator of oocyte quality (24).

**Figure 1:**
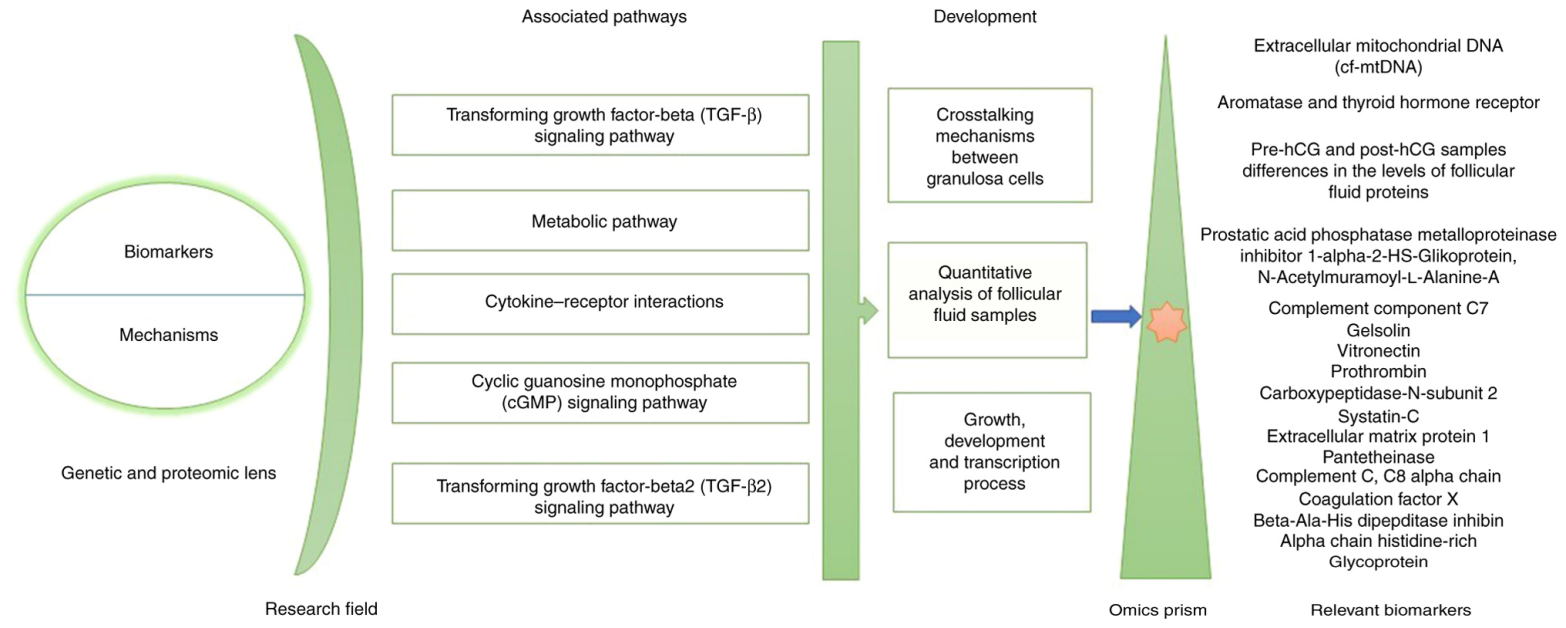
Diagram illustrating the genetic, epigenetic, and proteomic influences on the granulosa cells in follicular fluid and their associated pathways according to developmental mechanisms and transcription process with relevant biomarkers.

Granulosa cells are involved in various ovary-related diseases, including polycystic ovary syndrome (46). The interaction between granulosa cells and theca cells is crucial for early progesterone synthesis (47). Granulosa cells express specific biomarkers and receptors, such as aromatase and thyroid hormone receptors, highlighting their functional importance (48). Despite their physiological importance, granulosa cells and associated structures, such as the corona cumulus, are often discarded as waste during IVF treatments. However, their potential alternative applications or effects as waste have not been extensively investigated. Culture media enriched with various components used in IVF treatments support the growth and maturation of these cells and the oocyte (49). Further research into the potential use or analysis of these excreted components could enhance the understanding of reproductive biology. A summary of genetic, epigenetic and proteomic influences on granulosa cells in follicular fluid and their associated pathways according to developmental mechanisms and transcription process with relevant biomarkers is provided below and is illustrated in Fig. 1.

### Genetic research on granulosa cells in follicular fluid

The transcription information from granulosa cells, which are the somatic cells most adjacent to the oocyte, may mirror the developmental competence of the associated oocyte. Therefore, comparing the follicular fluid microenvironment and granulosa cell gene expression, particularly in patients with a poor ovarian response treated with various ovarian stimulation methods is critical for understanding the effects of different treatments on follicular physiology (5).

At present, exogenous gonadotropin (Gn) is used as a required drug for ovarian stimulation in ART by affecting the way granulosa cells, follicular fluid and oocytes interact, which in turn promotes follicular growth. In the study by Liu *et al* (50), the transcriptome of the granulosa cell was analyzed to determine whether Gn stimulation may potentially cause meiotic mistakes in human oocytes following natural and Gn stimulation cycles.

### Proteomic research on granulosa cells in follicular fluid

To gain a better understanding of the intrafollicular environment during oocyte maturation, Zamah *et al* (51) performed a proteomic analysis of follicular fluid from anonymous oocyte donors undergoing IVF oocyte retrieval. As a result, a total of 742 follicular fluid proteins were identified in healthy ovum donors, which included 413 previously unnotified ones. These mentioned proteins belong to various functional groups, such as insulin growth factors and its binding protein families, immunity, growth factors, anti-apoptotic proteins, receptor signaling and matrix metalloprotease-associated proteins. Moreover, a quantitative analysis of follicular fluid samples among the women with matched ages and between the pre-hCG and post-hCG samples indicated the vital differences in the levels of 17 follicular fluid proteins, which play a role in inflammation, cell adhesion and protease inhibition processes (Fig. 1) (51).

The study by Al-Saleh *et al* (52) examined the differences in hormone and cytokine levels between the follicular fluids of the mild ovarian stimulation and conventional groups. As a result, the follicle stimulating hormone, prolactin and progesterone levels in the follicular fluid were significantly lower in the mild ovarian stimulation group compared to the conventional one (52). Furthermore, the cytokine concentrations in the mild group were significantly higher in TGF-β2 and lower in growth differentiation factor-9 (GDF-9), while the bone morphogenic protein-15 (BMP-15) levels were comparable (Fig. 1) (33).

### Oxidative stress research on granulosa cells in follicular fluid

Reactive oxygen species (ROS), such as superoxide anion, hydroxyl radicals and hydrogen peroxide, are produced during mitochondrial electron transport for energy production, and are essential for controlling follicular growth, oocyte maturation, ovulation, fertilization, embryo implantation and fetal development (53). A surge in luteinizing hormone and neovascularization within the follicle during ovulation provides essential stimulation for follicular rupture and oocyte maturation, which in response stimulates the production of ROS (54).

When there is an imbalance between oxidation and the antioxidant system, ROS can oxidatively damage DNA, proteins and lipids either directly or indirectly and it can lead to gene mutations, protein denaturation and lipid peroxidation (55). Al-Saleh *et al* (52) indicated that oxidative stress biomarkers, including 8-oxo-2'-deoxyguanosine, total antioxidant capacity and malondialdehyde may be useful tools for assessing clinical features in patients undergoing IVF due to their association with reproductive hormones and pregnancy outcomes in ART. As aforementioned, follicular development and oocyte maturation are strongly influenced by the interaction between the oxidative, antioxidative system and metabolic products within a follicular fluid. Furthermore, this fluid is appropriate for determining the levels of oxidative stress in the follicular milieu and, as a result, the developmental potential of oocytes as it is simple and non-invasive to acquire (56). The complex role of oxidative stress, which may have undetermined advantageous functions in some circumstances, may help to elucidate the mechanisms through which oxidative stress can improve oocyte quality and result in normal fertility if the level of ROS in the cell is at an appropriate level (57).

## 6. Research on the cumulus-oocyte complex in follicular fluid

### Genetic research on the COC in follicular fluid

Cumulus cells (CCs) from follicles with varying diameters exhibit variations in gene expression related to metabolic processes, cell differentiation, and adhesion, according to one study's genetic ontology analysis. Additionally, research has discovered genetic biomarkers expressed in CCs that can predict the developmental competence of oocytes and provide a non-invasive manner to evaluate the quality of oocytes (58). Gene expression in CCs has been linked to embryonic development and pregnancy, suggesting a fundamental role of cumulus cell pathways (PAP1, RAS and ErbB pathways) in these processes (59).

In the study conducted by Su *et al* (60), oocytes were shown to regulate metabolic activities in CCs by upregulating the expression of certain genes that encode amino acid transporters and enzymes essential for oocyte-deficient metabolic processes. CC-associated genes, such as hyaluronan synthase homolog, prostaglandin-endoperoxide synthase 2 (Ptgs2) and TNF-stimulated gene 6 (Tnfsg6) have been shown to be abundant in CCs, which has implications for cumulus growth and subsequent embryonic development (61). Ligand-encoding genes with specific expression in oocytes or CC have been linked to biological functions, possibly linked to the coordinated formation of transzonal projections from CC that reach the oocyte membrane (62). The expression of expansion-associated genes, such as Ptgs2 or cyclooxygenase-2, TNF-alpha-induced protein 6 (Tnfaip6) and hyaluronan synthase 2 (Has2) in CCs is essential for the synthesis and stabilization of the extracellular matrix by CCs, and is crucial for cumulus expansion and ovulation (Tables I and II) (61). Furthermore, the downregulation of the Wnt signaling pathway in dysmature CCs was identified as a marker for assessing oocyte quality, demonstrating the importance of gene expression in CC as an indicator of embryonic development (Tables I and II) (63).

The expression of certain genes in CCs has been identified as potential markers for oocyte and subsequent embryo quality. The study by Uyar *et al* (6) demonstrated that the expression levels of genes in CCs are related to oocyte maturation, fertilization and embryo quality. In particular, the expression of Has2, Ptgs2 or cyclooxygenase-2 and gremlin1 in CCs was shown to be associated with oocyte and embryo quality (Tables I and II) (6). Akino *et al* (63) used next-generation sequencing to analyze gene expression in immature CCs, revealing the downregulation of the Wnt signaling pathway as a marker for determining oocyte quality. Furthermore, it has been demonstrated that the expression of certain genes in CCs is associated with embryonic developmental competence, suggesting their potential as markers for oocyte quality (Tables I and II) (64).

### Proteomic research on the COC in follicular fluid

Several proteins essential for the organization and function of the cumulus matrix have been identified, including pentraxin 3 (PTX3), TNF-induced protein 6 (TNFAIP6), BMP15 and GDF9. Following the pre-ovulatory luteinizing hormone, these proteins play a role in cumulus expansion, oocyte-cumulus cell contact and cumulus cell function control (65). Furthermore, proteins secreted by CCs have been shown to form ligand-receptor pairs that transmit paracrine signaling between the oocyte and CCs, demonstrating the complex communication and regulatory networks between these two components (62). PTX3 and TNFIP6 were identified as key components of the cumulus matrix required for cumulus expansion and IVF (Tables I and II) (65). Inter-α-trypsin inhibitor, TNFAIP6 and PTX3 were identified as proteins required for the formation and stability of the COC matrix (Tables I and II) (66). Protein levels of phospho-H2AX, breast cancer susceptibility gene 1, ataxia-telangiectasia mutated, meiotic recombination 11 homolog A and radiation repair gene were previously significantly increased in aging CCs, suggesting that these proteins are involved in the aging process (Tables I and II) (67).

### Oxidative stress research on the COC in follicular fluid

The presence of CCs during IVF has been shown to protect the oocyte against oxidative stress, thus improving initial division and subsequent development. The exposure of oocytes to hydrogen peroxide has been shown to result in oocyte death and the blockade of the first division, highlighting the protective role of CCs against oxidative stress during fertilization (68). Moreover, oxidative stress has been associated with fragmented DNA, poorer embryological development and increased miscarriage rates (Table I and II) (69).

## 7. Conclusion and future perspectives

The advent of non-invasive fluid biopsies has transformed the landscape of medical diagnostics and reproductive health, providing a more patient-friendly alternative to traditional biopsy methods. By leveraging biomarkers, such as EVs, circulating tumor cells and cfNAs, researchers have expanded the potential for early disease detection, prognostic evaluation, and personalized therapeutic strategies. These innovations have been particularly impactful in oncology, where liquid biopsies offer a means to detect tumor-derived genetic material and monitor treatment responses in real time. Additionally, in reproductive medicine, the ability to analyze cfDNA and cfRNA from embryo culture media has opened new avenues for non-invasive PGT, reducing the need for embryo biopsies and mitigating potential risks to embryo viability.

Despite these advancements, several challenges remain to be addressed before non-invasive fluid biopsies can become routine in clinical practice. The sensitivity and specificity of these methods vary across different conditions, necessitating the further refinement of isolation and detection techniques. Standardization remains a critical issue, as differences in sample collection, processing and analytical methodologies can lead to inconsistent results. Moreover, the low concentration of biomarkers in some fluids, such as ctDNA in early-stage cancer, poses a significant limitation, requiring improvements in enrichment and amplification technologies to enhance detection accuracy.

In reproductive health, while non-invasive methods such as the analysis of cfDNA in embryo culture media shows promise, their clinical utility remains under debate. The presence of maternal contamination, the accuracy of chromosomal assessments and the reproducibility of findings across different patient populations warrant further investigation. Additionally, the clinical application of EVs as biomarkers in fertility and pregnancy-related complications is an emerging field that requires extensive validation through large-scale studies.

Looking ahead, future research is required to focus on integrating multi-omics approaches, combining genomics, transcriptomics, proteomics and metabolomics, to develop more comprehensive and reliable diagnostic tools.

In conclusion, non-invasive fluid biopsies hold immense potential to revolutionize disease diagnosis, treatment monitoring and reproductive medicine. While significant progress has been made, continued advancements in technology, standardization and clinical validation are essential for these approaches to become fully integrated into mainstream medical practice. With ongoing innovation, non-invasive sampling techniques could ultimately lead to a paradigm shift in personalized medicine, offering safer, more efficient and widely applicable diagnostic solutions for a range of medical conditions.

## Figures and Tables

**Table I:** Overview of key non-invasive biomarkers relevant to IVF, listing their biological sources and summarizing their clinical implications.

Biomarker	Source	Clinical implications
Cell-free DNA (cfDNA)	Follicular fluid, embryo culture media	Indicates oocyte and embryo quality, apoptosis, and developmental competence; non-invasive genetic testing; lower levels linked to higher pregnancy rates.
Cell-free mitochondrial DNA (cf-mtDNA)	Follicular fluid, embryo culture media	Associated with cellular distress, apoptosis, and embryo fragmentation; lower levels linked to higher embryo viability.
Extracellular vesicles (EVs)	Uterine fluid, embryo culture media, endometrial secretions	Mediate intercellular communication; contain proteins, lipids, and nucleic acids; biomarkers for implantation, endometrial health, and embryo viability.
MicroRNAs (miRNAs, sncRNAs)	Follicular fluid, embryo culture media, endometrial exosomes	Regulate gene expression related to implantation and embryo development; non-invasive markers for embryo health and implantation potential.
Proteins (PTX3, TNFAIP6, BMP15, GDF9, MMPs, VEGF, FSH, LH, hCG)	Follicular fluid, cumulus cells, embryo culture media	Involved in cumulus expansion, oocyte maturation, embryo development, and ovarian response; markers for oocyte and embryo quality.
Oxidative stress markers (MDA, 8-OHdG, AOPP, TAC, SOD, GPx, GST, thiol groups)	Follicular fluid, cumulus cells	Reflect oxidative stress status; associated with reproductive hormones, embryo quality, and IVF outcomes.
Metabolites (glucose, lactate, glycerophosphocholine, androsterone sulfate, elaidic carnitine)	Follicular fluid, embryo culture media	Indicate metabolic activity, ovarian response, and predict clinical outcomes (pregnancy, miscarriage, live birth rates).
Gene expression profiles	Granulosa cells, cumulus cells, uterine fluid	Predict oocyte and embryo quality, endometrial receptivity, and pregnancy outcomes; used for personalized embryo selection.
Immunologic markers	Follicular fluid, blood, endometrial tissue	Indicate immune status, inflammation, and endometrial receptivity; potential predictors of implantation success.
Antioxidant enzymes (glutathione peroxidase, catalase, SOD)	Follicular fluid, cumulus cells	High levels are linked to better oocyte and embryo quality; protect against oxidative damage.
Time-lapse Imaging/morphokinetic parameters	Embryo culture media (image analysis)	Non-invasive assessment of embryo developmental kinetics and morphology; associated with live birth rates.
Transcriptomic biomarkers	Uterine fluid, endometrial secretions	Predict endometrial receptivity and window of implantation; guide timing of embryo transfer.
Seminal plasma biomarkers (cfDNA, proteins, metabolites)	Semen	Non-invasive assessment of male fertility, sperm retrieval potential, and ART outcomes.

The information presented in the table was obtained from previous studies (6,61,63-67,69). The biomarkers included in the table range from cell-free nucleic acids and extracellular vesicles to proteins, metabolites, oxidative stress markers, and gene expression profiles. sncRNAs, small non-coding RNAs; PTX3, pentraxin 3; TNFAIP6, tumor necrosis factor alpha-induced protein 6; BMP15, bone morphogenetic protein 15; GDF9, growth differentiation factor 9; MMPs, matrix metalloproteinases; VEGF, vascular endothelial growth factor; FSH, follicle stimulating hormone; LH, luteinizing hormone; hCG, human chorionic gonadotropin; MDA, malondialdehyde; 8-OHdG, 8-oxo-2'-deoxyguanosine; AOPP, advanced oxidation protein products; TAC, total antioxidant capacity; SOD, superoxide dismutase; GPx, glutathione peroxidase; GST, glutathione S-transferase.

**Table II:** Cumulus-oocyte complex in genetics, proteomics and oxidative stress research.

A, Genetic research on the COC in follicular fluid		
Key genes	Functional roles	(Refs.)
Hyaluronan synthase homolog prostaglandin-endoperoxide synthase 2, TNF-stimulated gene 6 (Hsh, Ptgs2 and Tnfsg6)	Genes affecting cumulus growth and subsequent embryonic development	(61,63)
Prostaglandin synthase-2 or cyclooxygenase-2, tumor necrosis factor alpha-induced protein and hyaluronan synthase 2 (Ptgs2, Tnfaip6 and Has2)	Cumulus expansion and ovulation	(61)
Hyaluronan synthase 2, prostaglandin synthase-2 or cyclooxygenase-2, and gremlin1 (Has2, Ptgs2 and Grem1)	Oocyte and embryo quality	(6)
B, Proteomic research on the COC in follicular fluid		
Structural proteins	Roles in the cumulus matrix	(Refs.)
Pentraxin 3, tumor necrosis factor-induced protein 6, bone morphogenetic protein 15 and growth differentiation factor 9 (Ptx3, Tnfip6, Bmp15 and Gdf9)	Organization and function of the cumulus matrix	(65)
PTX3 and TNFIP6	Key components of the cumulus matrix required for cumulus expansion and *in vivo* fertilization	(65)
Inter-Α-trypsin inhibitor (Iαi or ITI), tumor necrosis factor-induced protein-6 (TSG6 Or TNFIP6) and pentraxin 3 (PTX3 Or TSG14)	Proteins required for the formation and stability of the COC matrix	(66)
Protein levels of phospho-H2AX, breast cancer susceptibility gene 1 (BRCA1), ataxia-telangiectasia mutated (ATM), meiotic recombination 11 homolog A (MRE11) and radiation repair gene (RAD51)	Significantly increased in aging CCs, suggesting that these proteins are involved in the aging process	(67)
C, Oxidative stress research on the COC in follicular fluid		
Oxidative stress biomarkers	Impact for oocyte development	(Refs.)
Malondialdehyde (MDA), 8-Oxo-2'-Deoxyguanosine (8-OHdG), Advanced Oxidation Protein Products (AOPP), Total Antioxidant Capacity (TAC), Superoxide Dismutase (SOD), Glutathione Peroxidase (GPx), Glutathione S-transferase (GST), Thiol Groups	Fragmented DNA, poorer embryological development, and increased miscarriage rates	(69)

COC, cumulus-oocyte complex; CCs, cumulus cells.

## Data Availability

Not applicable.
